# Mixed Reality as a Digital Visualisation Solution for the Head and Neck Tumour Board: Application Creation and Implementation Study

**DOI:** 10.3390/cancers16071392

**Published:** 2024-03-31

**Authors:** Nadia Karnatz, Michael Schwerter, Shufang Liu, Aida Parviz, Max Wilkat, Majeed Rana

**Affiliations:** 1 Department of Oral and Plastic Maxillofacial Surgery, Heinrich Heine University Hospital Düsseldorf, Moorenstraße 5, 40225 Düsseldorf, Germanyrana@med.uni-duesseldorf.de (M.R.); 2 Brainlab AG, Olof-Palme-Str. 9, 81829 München, Germany

**Keywords:** mixed reality, software platform, tumour board

## Abstract

**Simple Summary:**

Studies have shown that the introduction of multidisciplinary tumour boards can have a positive impact on the survival of cancer patients. By bringing together the different disciplines involved in the treatment of cancer patients, tumour boards provide an interdisciplinary approach to decision-making in the treatment of oncological diseases. Tumour boards have become an integral part of treatment planning. However, their preparation is time-consuming and labour-intensive. The multiplicity of sources and clinical systems makes common communication difficult and consumes a lot of resources. Mixed reality technology could provide the necessary information as an interactive user interface. This is a new digital holographic imaging technology that can generate virtual 3D objects in space from radiological sectional images. A mixed-reality-based software prototype will be developed to analyse whether and to what extent this technology is suitable as a platform for decision making in the head and neck tumour board.

**Abstract:**

The preparation and implementation of interdisciplinary oncological case reviews are time-consuming and complex. The variety of clinical and radiological information must be presented in a clear and comprehensible manner. Only if all relevant patient-specific information is demonstrated in a short time frame can well-founded treatment decisions be made on this basis. Mixed reality (MR) technology as a multimodal interactive user interface could enhance understanding in multidisciplinary collaboration by visualising radiological or clinical data. The aim of the work was to develop an MR-based software prototype for a head and neck tumour board (HNTB) to support clinical decision-making. The article describes the development phases and workflows in the planning and creation of a MR-based software prototype that were required to meet the multidisciplinary characteristics of a HNTB.

## 1. Introduction

The contemporary therapy of oncological diseases is increasingly complex and specialised. To standardise and improve the communication and interaction with physicians, case conferences can help to plan the appropriate therapies. However, patient-specific information has increased, and the preparation of such a tumour board is sometimes time-consuming and labour-intensive [1]. In addition, the requirements for the compilation and visualisation of clinical data and findings differ between disciplines [2,3]. The multitude of sources and clinical systems complicates interdisciplinary communication and requires many resources [4].

MR technology could provide information as a multimodal interactive user interface [5]. It is a digital holographic imaging technology that allows objects to be generated virtually in space and provides a spatial experience and interaction [6]. By combining the real world with virtual objects, medical data can be visualised and explored in a new and unique way [7].

However, the acceptance of technologies is often linked to multiple factors, such as a needs-based user interface, intuitive usability or the simplification of established workflows [8,9].

As a multimodal platform, a mixed-reality-based head and neck tumour board (MR-HNTB) could support interdisciplinary oncological treatment planning by providing relevant clinical, radiological and histopathological data on a single interface [10]. The unique selling point of MR technology is its immersive character, i.e., immersion in an expandable world, direct interaction with a three-dimensional (3D) object and immediate visual feedback [7,11,12,13]. This extended perspective enables, for example, topographical observations [14,15,16,17]. However, the question arises as to the added value of these new possibilities, as many of the advantages mentioned above can also be achieved with 2D simulations. The integration of virtual objects into one’s own physical environment is innovative for MR-based applications. The intuitive forms of visualisation and interaction lead to an expansion or supplementation of the presentation and communication of information [18,19].

Viewing authentic and responsive objects and interacting in free space allows the user to experience depth and perspective, thereby supporting understanding and a completely new relationship to 3D visualisation [20]. The promotion of knowledge acquisition through interdisciplinary exchange and personal experience are further potentials [21]. Through the development of MR-based software, informatics and clinical approaches should be used, which, on the one hand, allow a clinically practicable implementation of a tumour board and, on the other hand, support interdisciplinary understanding.

The aim of this work was, therefore, to develop a software prototype for a mixed-reality-based head and neck tumour board to support clinical decision-making.

## 2. Materials and Methods

### 2.1. Analysis of the Organisation of a Conventional HNTB

The HNTB of the University Hospital Düsseldorf was selected as a model for the implementation of the MR software. For this purpose, audits were carried out at the beginning of this study to analyse the necessary processes, quality standards and subject-specific requirements for the realisation of a HNTB. The following 3 sections have been defined: (1) preparation/registration, (2) execution and (3) documentation.

During the initial phase, the clinical, pathological and radiological findings are summarised in a designated software mask of the hospital information system (HIS) after completion of the tumour staging. This also serves as registration for the HNTB.

In the implementation phase of the HNTB, the patient’s case and reports are discussed based on the registration information. The presentation of the computer tomography (CT) scans or magnetic resonance imaging (MRI) scans is implemented via the image archiving programme by the radiologist. As a rule, 3D renderings of the radiological data are not presented, but merely 2D slices. Decision-making is based on the clinical findings or the registration that is accessed via the HIS. The presence of a radiologist, pathologist and radiotherapist/oncologist, as well as otolaryngologist and maxillofacial surgeon, is mandatory. 

Finally, the head of the HNTB documents the decisions including the basis for the decision (e.g., guideline, patient’s choice of therapy, individual therapy trial).

### 2.2. Definition of the Phases and Analysis of the Requirements for the Realisation of a MR-HNTB

The study was conducted from May 2022 to April 2023 at the University Hospital Düsseldorf. The project started with the definition of the timeline, tasks and content of the sections. The project was divided into three phases of work. The first phase, which was the analysis phase, aimed to define the quality standards and subject-specific requirements and was planned to last for three months. The second phase, which was the development phase, was planned to last for six months. During this phase, a software prototype for the realisation of a MR-HNTB was to be developed based on the audits from the analysis phase and feedback sessions. In the 3-month test phase, the software prototype was to be trialled and evaluated.

The interview guidelines were created based on the SPSS principle according to Helfferich (2009) to structure the content for analysis and test phase audits [22]. Open-ended questions were used for feedback sessions in the development phase. The transcripts of the audits and feedback sessions were analysed using MAXQDA software 2022 (Software for qualitative data analysis, 2022, VERBI Software. Consult. Sozialforschung GmbH, Berlin, Germany) for qualitative content analysis. To present the collected data in a compact form and analyse their content, we summarised them using inductive category formation following Mayring’s (2015) method and created a category system [23].

(1) Analysis phase

In analysing the requirements identified during the audits, as well as the literature review based on the requirements for a digital tumour board product described by Hammer et al., 2020, the potential software implementations in developing the MR software prototype were defined [24]. This is shown in Table 1.

The requirements were analysed with the help of anonymously transcribed interviews with regular HNTB participants from all mandatory disciplines (*n* = 7).

(2) Development phase

**Figure 1:**
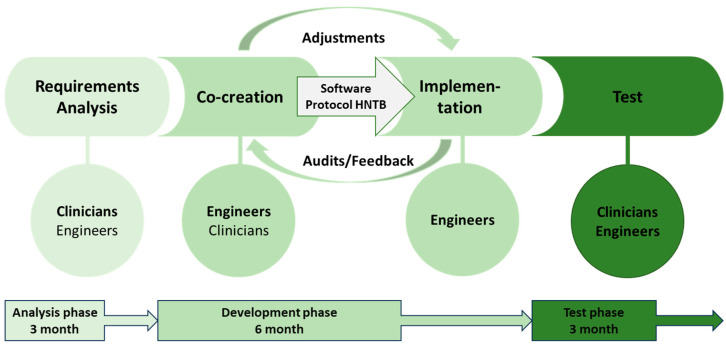
Process phases of software prototype development for a mixed-reality-based HNTB.

During the 6-month development phase, monthly structured feedback sessions were held on the requirements and their implementation. After each re-evaluation, an assessment took place within the development team to refine the MR software prototype based on the continuous user evaluations. The implementation of a MR-based software prototype in a simulated HNTB involved the following steps [Figure 1]:

- Formation of a multidisciplinary working group (3 clinicians, 2 engineers) for the development of a user interface according to the subject-specific requirements
- Regular assessments of the technical implementation
- Definition of the data that will be collected and visualised in the platform regarding the clinical case
- Regular adaptation of the platform to the requirements of the HNTB
- Definition of the workflow for the integration of the software into the HNTB

(3) Test phase

The software prototype was tested using 4 anonymised clinical patient cases in simulated MR-HNTBs with regular participants (*n* = 15) of the conventional HNTB in three sessions. One specialist member was mandatory. The cases originate from the Head and Neck Tumour Centre at Düsseldorf University Hospital. Their treatment had already been completed at the time of the study. After a ten-minute introduction to the hardware and software, the prepared cases were discussed. The MR-based user interface presented all the data from the medical history and treatment-relevant information in a MR environment. This was followed by a discussion of the cases.

In addition, the average preparation time for a case discussion in the MR-HNTB and in the conventional HNTB was recorded. When recording the preparation time for the conventional HNTB, all patient cases were included that were to be presented as part of two prospective HNTBs and met the inclusion criteria (*n* = 10); when preparing the MR-HNTB, patients whose treatment had already been completed were recorded in accordance with the inclusion criteria. Case discussions were prepared by an experienced resident with more than 4 years of experience or a senior physician with more than 5 years of experience in preparing an HNTB. The defined inclusion criteria were: (1) preoperative situation, (2) histologically confirmed squamous cell carcinoma of the oral cavity (primary tumour), (3) suspicious lymph nodes with a diameter of less than 1.5 cm and (4) no evidence of secondary carcinoma or metastasis in tumour staging. After completion of the simulation, the participants were audited to evaluate the software prototype. The transcription was anonymised.

The application prototype was developed as part of the project “Giga for Health Project: 5G Medical Campus” of the state of North Rhine-Westphalia/Germany in collaboration with the project partners Brainlab AG (Brainlab^®^, Munich, Germany) and the University of Düsseldorf.

### 2.3. Hardware and Software 

#### 2.3.1. Hardware

For the visualisation of the immersive MR content, head-mounted displays (HMD) with wireless transmission (Magic Leap 1, Plantation, FL, USA) were available as hardware technology. The Optical See-Through-HMD features simultaneous localisation and mapping (SLAM) capabilities [25]. Through the sensor-controlled registration in the environment, the position of the device in the physical space is recorded and continuously updated to achieve a spatial representation of the holographic information in the room or to fix virtually augmented objects in the real world. In addition, the Magic Leap 1 HMD has two fixed focal planes, one for content near the user and a second for room scale content (Table 2). The Magic Leap optics block 85% of real light and only transmit 15%, which is reflected in good colour contrast and lower light sensitivity [26].

**Figure 2:**
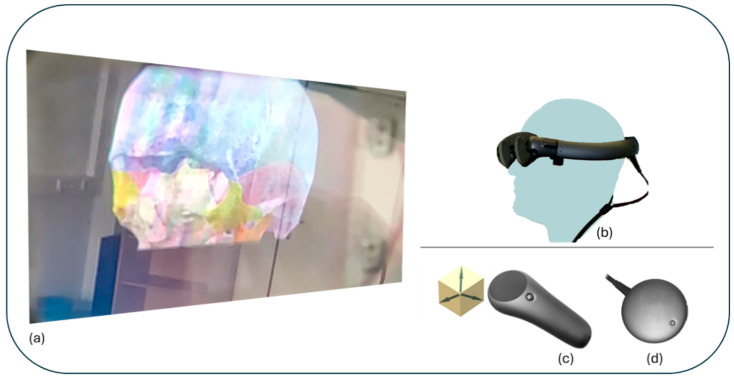
Magic Leap 1 Device (Plantation, FL, USA): (**a**) Visualisation of the immersive MR content. (**b**) Head-Mounted Display. (**c**) 6 DoF Controller. (**d**) Compute Pack.

The virtual content is navigated using controller-based manipulation with 6 degrees of freedom (6 DoF) [Figure 2]. The Magic Leap1 field of view is 50° diagonal, 40° horizontal and 30° vertical [26]. The implementation of MR technology on a portable computer enables mobility and spatial independence.

#### 2.3.2. Software

The MR-HNTB prototype software was created based on the MR viewer application from Brainlab Mixed Reality Viewer Version 5.3 (Brainlab AG, Munich, Germany) and adapted according to the specific requirements of the specialist departments and the quality standards of the HNTB. Technical and medical evaluations were conducted regularly during the software’s development to ensure a suitable user interface.

The Digital Imaging and Communications in Medicine (DICOM) data of the preoperative CT images were processed using Brainlab Elements software (Brainlab AG, Munich, Germany). The DICOM data of CT and MRI images were imported into the planning software, and relevant anatomical structures were segmented automatically (e.g., bone), semi-automatically (tumour) or manually (lymph nodes, critical structures). With the help of multimodal image fusion, additional information from the MRI scan could be included. CT examinations of the head and neck are routinely performed at Düsseldorf University Hospital with a slice thickness of 1 mm and contrast medium, MRI examinations with a slice thickness of 3 mm. Tumour staging includes mandatory radiological imaging such as head/neck CTs and thorax/abdominal CTs, as well as abdominal ultrasound examinations or head MRIs if necessary.

The MR software prototype has a developed import function that allows for the implementation of clinical findings and digitised histological sections. Thus, the histopathological findings from the surgical sampling, the clinical data from the medical history and the physical examination were available. The clinical data were prepared by a senior physician from the Department of Oral and Maxillofacial Plastic Surgery with more than 5 years of experience in ablative and reconstructive head and neck tumour surgery. The data were manually entered into a standardised portable document format (PDF) file.

The information from the datasets is transferred to the head-mounted MR device using a quick response (QR) code and visualised by the prototype of the viewer software.

### 2.4. Ethics

All data, except for the transcription notes from the feedback sessions during the development phase, were treated anonymously. The study was ethically reviewed and approved by the regional ethics committee of Heinrich Heine University Düsseldorf.

## 3. Results

### 3.1. Technical Realisation and Implementation of an MR-HNTB

#### 3.1.1. Preparation of the Radiological Cross-Sectional Imaging

**Figure 3:**
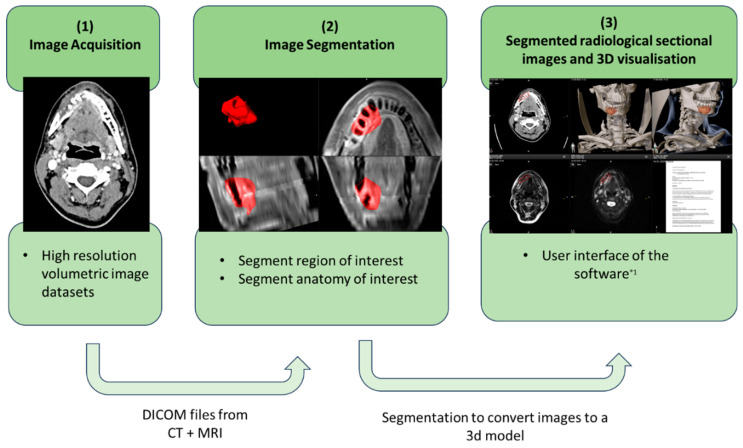
Workflow for the preparation of the radiological cross-sectional imaging; *^1^ Elements, Brainlab.

To use MR technology as a visualising and multimodal interface in the interdisciplinary exchange, the DICOM data of patients’ preoperative CT or MRI scans were imported into the planning software (Elements, Brainlab, Munich, Germany) to process them for 3D visualisation [Figure 3]. To ensure symmetry of the anatomy and reproducibility of the three-dimensional reconstruction, the CT slices are aligned in all dimensions (axial, coronal and sagittal) according to the Frankfurt horizontal plane before image fusion. With the help of image fusion, the multimodal or complementary information from the CT and MRI examinations can thus be utilised.

The fused images share the same coordinate system, and the segmentation results from one modality can be transferred to the other and vice versa. This allows the software to automatically segment structures from the most suitable image dataset.

The patient’s preoperative CT and MRI images were used to plan the MR-HNTB. After completing all the necessary pre-planning steps, the anatomical structures and the target tumour tissue were segmented from the image sets. Here, the MRI dataset can be used to identify the tumour tissue, and the CT dataset was used to define and visualise the patient’s osseous anatomy. The segmentation of the anatomical structures was based on an atlas-based algorithm. This allowed the skeletal structures to be segmented and used for further planning of the tumour resection or surgical reconstruction. In contrast, the tumour tissue was manually marked in an axial and sagittal single slice of the CT/MRI using the SmartBrush algorithm of the Brainlab software, and a 3D tumour volume was automatically created. If necessary, this was corrected manually accordingly in the slice images. Furthermore, analogous to the segmentation of the tumour, limiting structures can be colour-coded to assess and document the operability of the tumour or the planned resection margins. The aim of this planning step is a clearly defined anatomical representation of the patient’s anatomy or pathology.

#### 3.1.2. Preparation of Clinical Findings and Sectional Imaging

**Figure 4:**
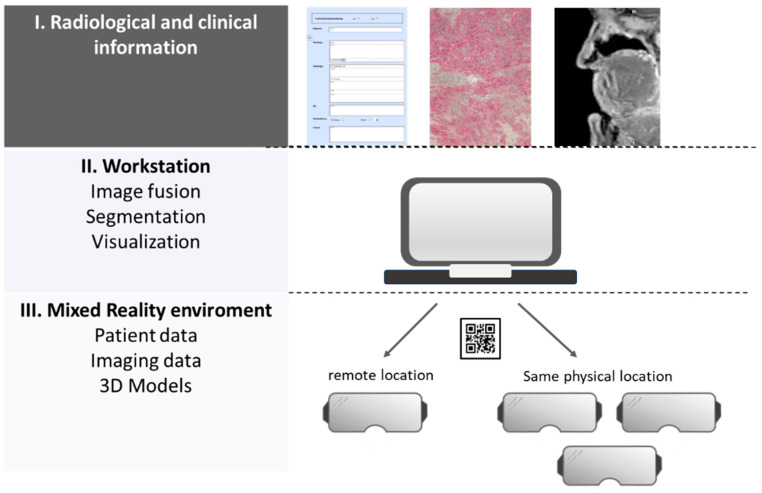
Workflow for a collaborative MR application.

**Figure 5:**
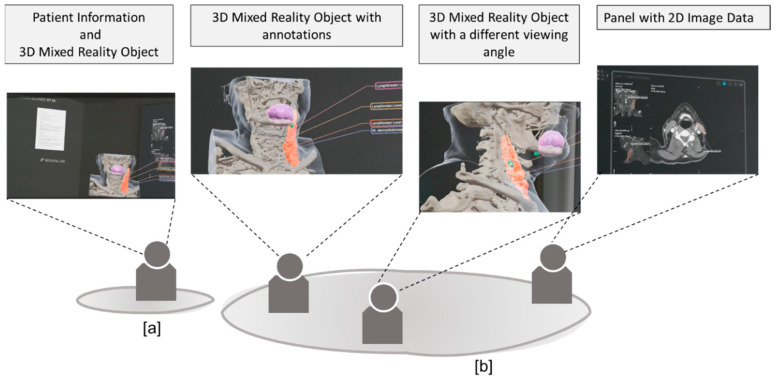
HNTB participants in the same MR environment with a different view of the same panels or 3D models (remote [**a**] or local [**b**] collaboration).

The clinical and histological findings and images were compiled manually in a specially created PDF template. The compiled information was then added to the radiological patient dataset. For each case discussion, there was a dataset that contained the necessary clinical, pathological and radiological information, as well as a 3D visualisation of the segmentation. The data content was transferred to the HMD via a QR code on the software interface and transmitted as an overlay in a collaborative MR environment. By using multiple virtual panels on the MR user interface, all clinical case information, illustrative images and radiological images were available to all HNTB participants [Figure 4 and Figure 5].

#### 3.1.3. Integration of Tools to Support Collaboration

The shared virtual platform enabled interactions with the objects and panels in real time with the help of the controller. At the same time, discussions between the participants were possible in the physical world. The use of 3D rendering in the sectional view and the provision of comments supported interdisciplinary communication. By integrating an audio tool into the software prototype, the spatial limitations of the HNTB participants could also be removed so that they were independent of their current location. The medical data were virtualised with the MR-based solution in a collaborative space and visualised at the remote user’s location. The participants could see how the remote participant interacted with the virtual objects. The HNTB participants were able to navigate and collaborate with the controller in the virtual space and provide auditory feedback.

### 3.2. Time Requirement for the Preparation of Case Discussions

The preparation of the case discussions showed that the preparation of the clinical cases for the MR-HNTB took more time than for conventional HNTB. The average preparation time for conventional HNTB (*n* = 10) was t^*1^ = 13 min (*SD* = 2.45), for MR-HNTB (*n* = 4) t^*2^ = 47 min (*SD* = 8.18).

The reason for this, apart from the additional time required to convert the data into the DICOM format, is the segmentation of the radiological sectional images as additional visual information. This work step is not necessary with conventional HNTB. In addition, the complexity of the case and the speed of processing the case review are influencing factors so that the data are not meaningful for the small number of cases. Another significant factor influencing the time required is the lack of an interface between the MR technology, the resulting manual input of all data and the familiarisation with new software.

### 3.3. Qualitative Evaluation of the Feedback Sessions and Audits 

#### 3.3.1. Qualitative Assessment of the Audits in the Analysis Phase

After evaluating the audits in the analysis phase, the results were structured according to Mayring’s qualitative content analysis (Table 3) [23].

This showed that in addition to the provision of all relevant findings on one interface, intuitive and user-friendly use of the software and hardware, as well as a structured, interdisciplinary tumour board process, were important to the participants. These results were in line with the requirements for a digital tumour board product postulated by Hammer et al. (2020) [24].

The following software implementations were summarised and prioritised according to the categories and subcategories:

1. Easy access to relevant findings
2. Creation of one presentation per case presentation
3. Integration of the various interdisciplinary requirements
4. User-friendly and intuitive user interface

#### 3.3.2. Qualitative Assessment of the Feedback Sessions in the Development Phase

Emerging issues with the software prototype or implementation were defined and discussed in the monthly feedback sessions. For example, open-ended questions were used to evaluate the structuring of content and technical workflows, the design of the user interface and the positioning of functions. When answering the questions, further ideas and suggestions emerged, which were added to the priority list as new functions and ranked. On this basis, the team prioritised core functions and developed ideas for improvements and solutions.

Three main categories were identified (Table 4): 

Issues in main category I were continuously developed and evaluated during the development phase until all members of the development team agreed that the result was satisfactory.

The main categories II and III had no priority and were, therefore, bypassed with temporary solutions (Issue II) or not considered during implementation (Issue III).

A permanent solution to the category “Issue II” would have been associated with a high consumption of resources, so the development team decided on a resource-saving implementation, which should not have a negative impact on the implementation of the test phase but cannot be regarded as a permanent solution. A project-related activity processing was created in consultation with the data protection officer at Düsseldorf University Hospital to ensure compliance with the data protection guidelines. As this was a software prototype, only the analysis of anonymised, retrospectively collected patient data was carried out. To ensure a stable data connection and due to the available hardware, the maximum number of participants was limited to 10. The subcategories of the “Issue III” category were not taken into account, as it would not have been technically or legally possible to implement them as part of this project.

#### 3.3.3. Qualitative Evaluation of the Audits during the Test Phase 

As in the audit phase, we conducted semi-structured interviews with the participants of the simulated mixed-reality-based HNTB using an interview guide. The interviews were evaluated and structured according to Mayring’s qualitative content analysis [23]. We divided the main categories into positive and negative feedback (Table 5).

**Figure 6:**
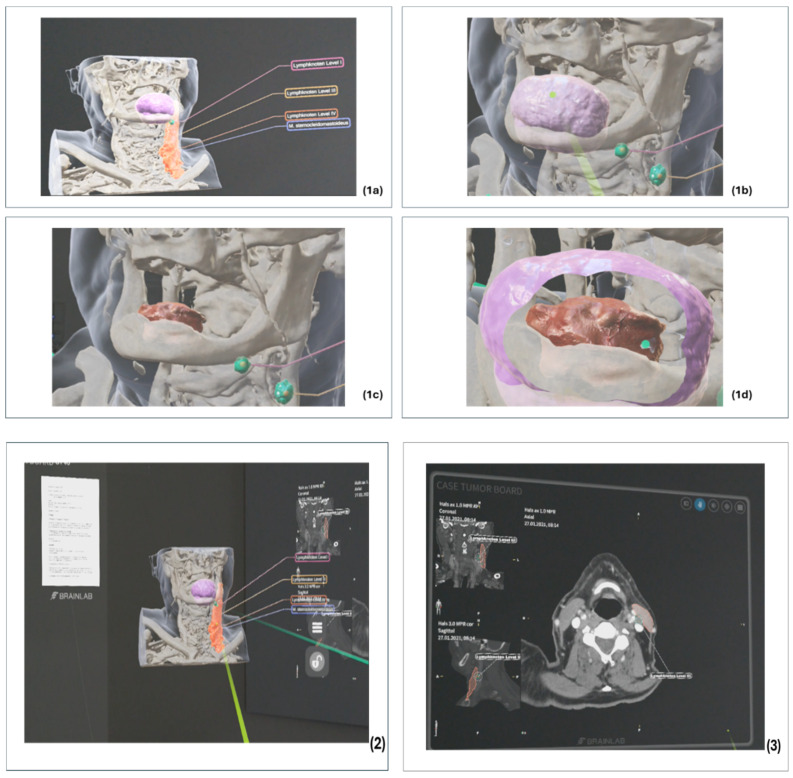
Examples of implementing the results of the feedback sessions in the development phase; (**1a**) MR view of a 3D model with annotations and colour coding of different structures (violet for safety margin, green for lymph nodes, orange for muscle); (**1b**) Different perspective on the same model after removal of the muscle; (**1c**) Different angle of view of the same model after removal of the safety margin with a view of the tumour; (**1d**) View of tumour and safety margin; (**2**) Overview of the software prototype interface with all relevant clinical and radiological information after integration of various file formats (PDF, DICOM) during interdisciplinary interaction/communication (green and turquoise pointer) (**3**) Radiological imaging in the MR environment with the segmented structures.

Due to the absence of a defined systematic process, in contrast to the established guidance in conventional HNTBs, and the use of a new interactive technology, disorganised and simultaneous interactions among several participants with virtual objects occurred repeatedly. This resulted in disorganisation and increased time expenditure. Furthermore, due to the absence of an interface with the electronic HIS, conventional information systems had to be used in case of uncertainties regarding case reports. To enhance interdisciplinary communication, the integration of standard transformation language (STL) files based on the planned reconstruction, immersive 3D visualisation of radiological data and annotations for additional information proved to be beneficial (Figure 6). 

**Figure 7:**
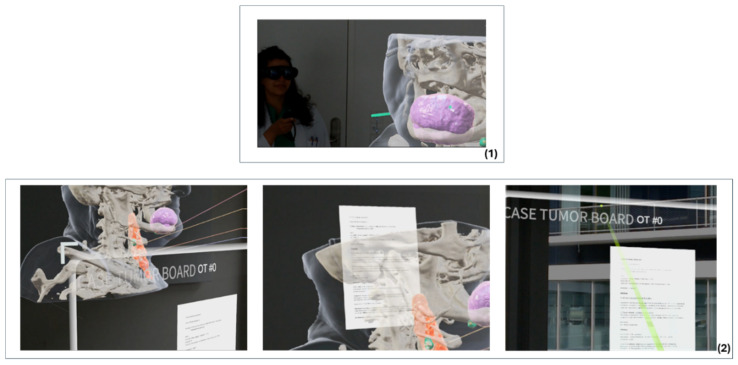
Scenes from the mixed-reality-based head and neck tumour board: (**1**) Screenshots from the observer’s MR headset, showing a participant’s exploration in a collaborative session; (**2**) Problems with simultaneous panel positioning.

It became evident that location-related factors, such as lighting conditions and the positioning of virtual objects, have an impact on the MR experience (Figure 7). Despite this, there was a high degree of agreement regarding clinical feasibility. The documentation revealed the greatest deficits in relation to MR technology. Since there was no way of documenting decision-relevant facts and decisions, the conventional system had to be used again. Structured documentation of case decisions is essential for validating tumour board decisions. Issues such as a lack of interface to other information or archiving systems were not taken into account by the development team from the outset (see Section 3.3.2) but were important factors for the test subjects in the test phase when evaluating the MR-HNTB in the interviews.

## 4. Discussion

The multidisciplinary tumour board is an evidence-based organisational approach to implement a more effective approach to tumour therapy for patients [28,29]. The provision of relevant information is important in order to plan interdisciplinary oncological treatment according to the guidelines and by consensus [30,31,32]. Against the background of increasing digitalisation in everyday clinical practice, we have developed an immersive 3D user interface to adequately visualise patient-related data in a simulated HNTB [33]. The software platform integrates a virtual 3D model of oncological patients, based on their CT or MRI scans, as well as other radiological and clinical data and illustrative images in a MR environment. An optimal understanding of all involved parties compared to the current practice, i.e., 2D visualisation on a computer screen, is made possible. The interdisciplinary endorsement of this technology, combined with the positive response of study participants to its potential clinical use, is a promising indicator for the future of data visualisation in conferences. This is in line with the findings of Janssen et al. (2018), who demonstrated that real-time data and images can improve patient coordination, even if they are not always optimally used by teams [34]. The limiting factors for clinical use are currently the manual, time-consuming collection of clinical information and the integration of a new technology into established structures.

In their study from 2023, Zhang et al. presented cloud-based software for conducting a tumour board in which the participants could act asynchronously [35]. This showed that there was a high level of agreement in the opinions of the medical experts. This shows how important it is to integrate new software into everyday clinical practice and to provide all relevant information. This is the only way to ensure guideline-based and appropriate decision making. An interface to existing digital hospital information systems or clinical image viewing software could be a possible step to improve the technological compatibility of MR-based technologies [34].

Considerable potential is seen in the discussion of complex oncological cases, as the location- and context-specific presentation of information improves interdisciplinary and specialist communication [36]. The use of 3D models or 3D representations for communication has already been the subject of several studies [37,38,39]. Kolecki et al. (2022) suggested the use of new VR and AR technologies to break new ground in clinical education [40]. Other studies have also shown that the colour visualisation of data is a crucial component in the evaluation and processing of information [41,42]. In the present study, these factors were also rated positively by the subjects of the MR-HNTB.

The possibility of physical movement, such as walking around the virtual object and physical interaction (e.g., stepping on the object [Figure 6(1d)]), also facilitates the spatial understanding of complex structures. Avoiding context and gaze shifts when recording this information promotes subject compliance [7,43]. This technology may be particularly useful in an era where tumour boards can be conducted using remote collaboration. Virtual rooms or the possibility of virtual co-presence represent an alternative to the traditional tumour board [44]. However, this technology with all its interaction and communication capabilities requires a strict leadership culture, as well as team management, discipline and appropriate procedures, as this is the only way to ensure effective and efficient interactive collaboration [45,46]. 

In this context, the difficulties of technological integration and the challenging issue of processing patient data in compliance with data protection regulations should be mentioned as obstacles to the routine use of MR technology in everyday clinical practice. The implementation of MR software in network-based inter- and intra-hospital traffic must be legally compliant with regard to the responsible handling of data and patient safety. The handling of patient data or the implementation of an IT infrastructure using secure network environments are complex and require a separate discussion of risks and benefits.

Despite the positive feedback from MR users on the possibilities of a MR-HNTB, further development should include a reassessment of user-friendliness with regard to adaptation to the requirements of the tumour boards of other disciplines. This could increase the acceptance and, thus, the adherence of tumour board participants [47,48].

In the field of conventional tumour board software solutions, several studies have already shown that virtualisation can optimise patient management [49]. In their observational study investigating the structuring processes and the implementation of a virtual multidisciplinary tumour board, Blasi et al. (2021) described how an efficient virtualisation and database system can potentially save time [50]. Thus, virtual multidisciplinary tumour boards are increasingly used to achieve high quality treatment recommendations across health regions, allowing the local multidisciplinary tumour board teams to expand and develop into a regional or national network of experts [51]. The use of MR-based tumour boards has particular potential for interdisciplinary collaboration in the context of remote collaboration and the creation of a shared virtual space. In addition to the small number of case studies used, the selection of subjects is important when evaluating the methods used in the study and the results. These were members of the HNTB at the University Hospital of Düsseldorf. The positive response in the qualitative evaluation of the test-phase audits can, therefore, be seen as evidence of the correct implementation of the quality requirements for the HNTB using MR technology. However, there was also a certain degree of scepticism about the technical requirements and their future feasibility. In addition to providing the software, the hardware must also be available in sufficient numbers to enable all professionals to participate. This requires a stable data connection and a secure network environment for real-time interactive collaboration.

With regard to the small number of cases, it should be noted that the segmentation processes described in Section 3.1 for the creation of patient-specific 3D models, including pathologies, require a considerable amount of time for manual processing. This is certainly a limiting factor in terms of potential clinical application. Most likely, this will become less important in the coming years due to technological advances in automated segmentation and registration software [52].

Although our focus has been on process, technology and resource issues, we are aware of the potential of using MR technology so that with appropriate development and the use of specific user interfaces, the technology could be more than just a viewer in clinical use. For example, registration and documentation should no longer be done manually. Other software solutions for the implementation of tumour boards already offer solutions for this. Nevertheless, the effort is worthwhile, as Specchia et al. (2022) and Hammer et al. (2020) have already reported that digital solutions for tumour boards can lead to an increase in efficiency during implementation without any loss of quality [53,54].

As the focus of this study was on qualitative analysis and evaluation of a potential clinical application, there were no measurable endpoints to report. The aim of the work was to assess the feasibility and potential benefits of an MR-HNTB. This may form the basis for further evaluations, such as a quality assessment of tumour board decisions depending on the visualisation of the data, a workflow assessment by quantifying the process steps required to prepare and conduct the tumour board session, or an assessment depending on the level of training and specialisation of the tumour board participants, and possibly a pilot in clinical use.

## 5. Conclusions

As part of this study, we developed a software prototype for a mixed-reality-based head and neck tumour board. It was shown that this can be a powerful tool for multidisciplinary collaboration in terms of factors such as the visualisation of clinical and radiological data on an intuitive platform. However, the use of MR technology is limited by the still existing deficits in the preparation and documentation of case discussions and the lack of interfaces with other information or archiving systems. Integration into everyday clinical practice is, therefore, likely to be difficult at present.

## Figures and Tables

**Table 1:** Requirements for a software prototype for a mixed-reality-based HNTB based on Hammer et al., 2020 [24].

Process or Quality Challenge	Technical Requirements
Visualisation of therapy-relevant information Creation of a single presentation to prevent clinicians from working on different user interfaces Standardisation of the visualisation workflow Better coordination and execution of tumour board meetings Integration of critical and essential information Demonstration of the multidisciplinary nature of discussions	System for the visualisation and presentation of tumour board cases Good usability and intuitive user interface Adaptable to current workflows Real-time acquisition of decision-making data

**Table 2:** Technical data overview Magic Leap 1 [26,27].

**Operating System**	Lumin OS
**Processor**	Nvidia Parker SoC
**GPU**	Nvidia Pascal 256 CUDA
**RAM**	8 GB
**Storage (ROM)**	128 GB
**Resolution**	1280 × 960 pro Auge
**Frame rate**	122 Hz
**Field of View**	50° diagonal, 40° horizontal and 30° vertical
**Eye tracking**	yes
**Weight**	316 g

**Table 3:** Summary of the analysis audits as a category system according to the inductive content analysis by Mayring [23].

Main Category	Subcategory
Structuring the tumour board	Management of the tumour board Case preparation Case presentation Interdisciplinary communication/interaction Documentation
Technical requirements/ Software requirements	Integration of standard file formats Intuitive use of the user interface Training in using the viewer software
Compliance with quality standards	Securing data protection Visualisation of radiological findings in sufficient quality
Providing the relevant information	Providing radiological data Providing clinical data Providing histological data Displaying data on a single user interface Structuring the user interface

**Table 4:** Summary of the development phase feedback sessions as a category system according to Mayring [23].

Main Category	Subcategory
Issue I	Light incidence/positioning Possibility of surface structuring (standardised presentation of recurring structures) Case presentation: effective and targeted use of medical information Real-time presentation Integration of different file formats Authenticity of the structures Intuitive use Interaction/Communication
Issue II	Stable data connection Setting the maximum number of participants Data protection guidelines
Issue III	Information Technology (IT) interfaces with HIS Providing further information IT interface with picture archiving and communication system IT interface with other information systems

**Table 5:** Summary of the test phase audits as a category system according to Mayring [23] (*) were not taken into account in the development of the software prototype.

Main Category	Subcategory
Positive feedback	Process Using medical information effectively and efficiently Increased interdisciplinary understanding through 3D visualisation Software Intuitive user interface Standardised surface structuring of the pathology through texture/colour Fulfilled expectations of prototype (clinical feasibility) Hardware Allows participants to move freely around the room
Negative feedback	Process Preparation time for case presentation Lack of an approach to systematic management of the HNTB (as simultaneous processing of the user interface is possible) Software Simultaneous processing of the user interface Technological dependence No possibility of documentation Lack of transparency of information (recourse to the HIS if information is missing) * Lack of evaluated workflow regarding data security hardware Technological dependence Hardware wear comfort Preparing the technical requirements Dependence of the light positioning

## Data Availability

The data are contained within the article.
